# Brain-Inspired Intelligent Systems for Daily Assistance

**DOI:** 10.1155/2019/7597839

**Published:** 2019-06-18

**Authors:** Anastassia Angelopoulou, Jose Garcia-Rodriguez, Epameinondas Kapetanios, Peter M. Roth, Kenneth Revett

**Affiliations:** ^1^University of Westminster, London, UK; ^2^University of Alicante, Alicante, Spain; ^3^Graz University of Technology, Graz, Austria; ^4^HCL Infosystems Ltd., Boston, MA, USA

The fields of machine learning and cognitive computing have been in the last decade revolutionised with neural-inspired algorithms (e.g., deep ANNs and deep RL) and brain-intelligent systems that assist in many real-world learning tasks from robot monitoring and interaction at home to complex decision-making about emotions and behaviours in humans and animals. While there are remarkable advances in these brain-inspired algorithms and systems, they need to be trained with huge data sets, and their results lack flexibility to adapt to diverse learning tasks and sustainable performance over long periods of time. To address these challenges, it is essential to gain an analytical understanding of the principles that allow biological inspired intelligent systems to leverage knowledge and how they can be translated to hardware for daily assistance and practical applications. This special issue brings researchers from interdesciplinary domains to report their latest research work on algorithms and neural-inspired systems that flexibly adapt to new learning tasks, learn from the environment using multimodal signals (e.g., neural, physiological, and kinematic), and produce autonomous adaptive agencies, which utilise cognitive and affective data, within a social neuroscientific framework. In this special issue, we have selected five papers out of fourteen high-quality papers after a careful reviewing process, which brings the acceptance rate to 35.7 percent. The five papers are representative of the current state-of-the-art in this area.

E. Cruz et al. present a robotic system for monitoring and interacting with people affected by cognitive diseases by successfully integrating object recognition, activity recognition, localisation, and navigation methods to remember and help the patients to perform their daily tasks. The proposed methodology involves the implementation of an Object Recognition Engine (ORE) responsible to detect a certain object based on the Inception ResNet V2 architecture, a classic convolutional neural network (CNN) scheme over region-based convolutional neural network (R-CNN) architectures to avoid false detections. Furthermore, a Behaviour Recognition Engine (BRE) based on the OpenPose algorithm is then used to properly recognise the user's behaviours in different rooms and in different houses. The proposed system was tested successfully on the human-like robot Pepper where additional information was localised on the robot such as how the robot adapts to new environments (*e.g., furniture in a room has been rearranged*) based on a semantic localisation system (SLS) and how the robot navigates from one room to another based on expert systems that compute the path from the current room to the target. The proposed methodology shows competitive and consistent results.

On the contrary, H. Ponce et al. present a methodology inspired by nature-control systems for Robots in Assisted Living (RAL) navigation systems using vision-based strategies based on Hermite optical flow (OF) and convolutional neural networks (CNNs). This integrated system uses Hermite OF for obstacle motion detection and CNN for obstacle distance estimation. The authors estimate the distance to mobile and fixed objects using a monocular camera instead of RGB-D sensors that provide depth maps of the scene. The advantage of this method is that it needs less data for training the distance estimator and no training data to compute the OF field. For experimental purposes, the authors used the robot simulator V-REP that recreates the conditions of the physical world quite accurately. The proposed methodology strengthens the hypothesis that a bioinspired OF method, a CNN technique for distance inference, and an artificial organic controller (AOC) can simulate a cognitive vision strategy in a dynamic environment.

S. Guan et al. propose first a subject-specific decision tree (SSDT) classification framework and second a data reduction method to distinguish multiclass motor imagery (MI) using electroencephalogram (EEG) signals for the brain-computer interface (BCI) based on the manifold of covariance matrices in Riemannian perspective. The goal of the SSDT classification framework is to separate the two MI tasks with the highest recognition rate and at the same time to enhance the classification accuracy. This is achieved by calculating in the tree a filter geodesic minimum distance to Riemannian mean (FGMDRM) in order to reduce the classification error. This method compared to other well-established methods in the literature performs better when EEG signals of the fixed frequency segment (8–30 Hz) are processed. The goal of the data reduction methodology, which includes a feature extraction and a classification algorithm, is to reduce in a nonlinear fusion the dimension of vectors in the Riemannian tangent plane and to classify different types of MI tasks based on the k-nearest neighbor (KNN) algorithm. The feature extraction algorithm, named “SJGDA,” combines semisupervised joint mutual information (semi-JMI) with general discriminant analysis (GDA). The proposed data reduction method performs better than semi-JMI and GDA on different datasets with higher recognition rates.

F. Gomez-Donoso et al. present a methodology that complements Ambient Assisted Living (AAL) environments, which are composed of cameras fixed to the ceilings of the environment, by integrating a domestic robot, namely, Pepper, which roams through the home to detect dangerous areas and movable (nonstatic) objects that the fixed cameras are not able to detect. In addition, the proposed system detects inhabitants' fall event and their extended stay in a particular area of the home. The proposed pipeline implements three object detection and tracking algorithms. First, a multimodal evolutionary algorithm based on a set of single agents is used to detect and track people behaviour in 3D. Second, an Obstacle over the Ground Tracker (OGT) based on the Random Sample Consensus (RANSAC) model-fitting algorithm is coupled to the robot to assist it to detect fixed and moving objects and obstacles above the ground level. Finally, a Superficial Object Detector (SOD) algorithm is used to build a more comprehensive map of the potentially dangerous areas by recognising objects like wall or floor sockets that are too small to be sensed by the 3D camera. This is achieved by parsing the colour images captured by the camera of the robot to a region-based convolutional neural network (R-CNN) which returns the bounding box and the category of the objects. The performance of each algorithm is validated by a serious of experiments in real-world environments.

Finally, Y. Hou and S. Chen present a system for characterising emotions using EEG signals, where in particular four classes of emotions (i.e., happy, sad, calm, and angry) should be distinguished. To this end, they induced these emotions by music stimuli (using 20 music passages in each music emotion group) and recorded the EEG signals of the subjects using 12 electrodes. From the obtained signals, 27 different features have been extracted, which have been used for further data analysis. In this way, the most valuable features have been identified by using the CFS method. In addition, it was revealed that C4.5 is more effective for emotion classification based on EEG signals than other widely used methods such as LDA or SVM.

